# The Impact of Social Distancing Due to COVID-19 on Activities of Daily Living in Parkinson’s Disease

**DOI:** 10.3390/healthcare11121688

**Published:** 2023-06-08

**Authors:** Patricia Sánchez-Herrera-Baeza, M.ª Pilar Rodríguez-Pérez, Gemma Fernández-Gómez, Nerea Bustamante-Palomo, Sergio Serrada-Tejeda, Paula Obeso-Benítez, Matilde Morales-Cabezas, Rosa M. Martínez-Piédrola, Marta Pérez-de-Heredia-Torres

**Affiliations:** Department of Physical Therapy, Occupational Therapy, Rehabilitation and Physical Medicine, Rey Juan Carlos University, Avenida de Atenas s/n, CP 28922 Madrid, Spain; patricia.sanchezherrera@urjc.es (P.S.-H.-B.);

**Keywords:** Parkinson’s disease, COVID-19, social isolation, activities of daily living, manual dexterity

## Abstract

Introduction: To explore the impact of the lockdown and social distancing measures, applied for one year, due to the COVID-19 pandemic on Activities of Daily Living in patients with Parkinson’s disease, as well as to determine the association between daily performance and tasks requiring more manipulative dexterity. Methods: Data collection was carried out between 18 January and 22 March 2021 through telephone interviews. Patients were recruited from associations of patients with Parkinson’s disease in Spain. A questionnaire was designed including items from standardized tools of the Activities of Daily Living Questionnaire to measure the level of independence and from the Dexterity Questionnaire for manipulative dexterity. Results: There were 126 participants aged 36–89 years, 58% of whom were male. The results of our study reveal a significant decline in almost all the ADLs assessed. There is a moderate correlation between the degree of dependence in ADLs and the difficulty in performing activities requiring manipulative dexterity. Conclusions: Social isolation related to the COVID-19 pandemic and its consequences may have contributed to an increase in the deterioration of manipulative ability, leading to a loss of ability to perform ADLs. These results show specific needs to be considered in the rehabilitation treatment of these patients.

## 1. Introduction

Parkinson’s disease (PD) is a chronic disease caused by the degeneration of dopaminergic cells and characterized by motor symptoms, such as bradykinesia or rigidity, as well as a wide range of non-motor symptoms [1]. Worldwide, there are 10 million people diagnosed with PD [2]. People with PD suffer limitations in the performance of Activities of Daily Living (ADLs), affecting the performance of activities that require coordination and balance, such as walking, reaching, grasping, manipulation and manipulative dexterity, as well as cognitive activities, such as speaking and especially those requiring executive functions. It is these abilities that largely determine the degree of independence in ADLs [1,2].

Coronavirus disease 19 (COVID-19) was declared a global pandemic by the World Health Organization in March 2020 [3], and measures of social isolation related to COVID-19 affected the daily routines and activities of the entire population [4,5], especially people with chronic diseases [4,5,6]. Rehabilitation and clinical services were temporarily closed, and non-urgent consultations were postponed [4,5]. Thus, the approach to PD was affected both by the suspension of treatments and because routine adjustments to medication were carried out with difficulty [7].

Previous research on the impact of confinement in this population has described a worsening of motor, emotional, cognitive, and communication areas in PD [8], which may even be sustained in the long term [5,9]. In this line, they associated reduced activity with increased global signs and symptoms of PD [10]. The motor symptoms particularly affected are bradykinesia, rigidity, tremor, dyskinesia, muscle cramps or spasms, and fluctuations [7,8]. Regarding the cognitive sphere, there is evidence that patients with Parkinson’s may have accelerated cognitive decline because of isolation, stress, and disruption of care, especially in relation to attention and memory [11]. Other areas affected are fatigue, anxiety, and depression [5], which, in turn, intensify motor and neuropsychiatric symptoms [7] and may also reduce the effect of dopaminergic medication [12].

COVID-19 confinement has altered the symptomatology and daily rhythm of patients with PD [4,11], but the impact of this worsening on ADLs has not yet been studied. In addition, after the end of the confinement, several social restrictions have been implemented and extended for one year. It is important to study how and what impact these prolonged restrictions have had on the functionality and ADLs of patients with PD [11,13,14].

The main objective of the study is to analyze changes in activities of daily living (basic and instrumental) of Parkinson’s patients during COVID-19 confinement and as a secondary objective, to identify whether there is a relationship between the level of independence in activities of daily living and the degree of difficulty in performing tasks requiring manual dexterity.

## 2. Materials and Methods

### 2.1. Study Design

A cross-sectional, both descriptive and correlational, design was used following the guidelines of the Strengthening the Reporting of Observational Studies in Epidemiology (STROBE) Checklist [15]. This study was approved by the ethics committee of ENM127/201301202101621. The collection, processing, and transfer of data were completed in accordance with the provisions of the Declaration of Helsinki [16] and current Spanish legislation on personal data protection.

### 2.2. Participants

Patients with PD were selected by contacting PD patient associations in Spain through the Spanish Parkinson’s Federation. Participants were recruited by using convenience sampling over a 3-month patient recruitment period. The estimated effect size for the main outcome measures established in the present work was 0.30. Considering a power of the statistical test of 0.80 and an alpha error of 0.05 for the correlation (point biserial model), a minimum of 82 subjects is required for the present work according to the G*Power software (V.3.1.9). The following selection criteria were established: patients (1) patients in stages II, III, and IV of the Hoehn and Yahr scale, >60% Schwab and England functionality scale, patients whose motor response to pharmacological treatment was stable or slightly fluctuating, (2) belonging to a Parkinson’s association in Spain, (3) perform conventional Occupational Therapy and Physiotherapy treatment (4) able to understand and respond to the survey in Spanish and (5) freely accepting participation in the study by signing the informed consent form. We excluded patients (1) previously diagnosed with COVID-19, (2) stages I or V of the Hoehn and Yahr scale, (3) unable to communicate verbally/non-verbally, (4) with cognitive difficulties in answering the questions required for the assessment and (5) who had some type of hearing impairment that cannot be corrected with hearing aids, making communication with the patient difficult.

### 2.3. Procedure

Data collection was conducted between 18 January and 22 March 2021. The survey was conducted by telephone, following the principles of confidentiality and voluntary participation [16]. At the time of the interview, due to the pandemic, the patients were not attending conventional rehabilitation treatment at the association.

A questionnaire was designed that included 6 socio-demographic variables, 3 clinical variables and others derived from standardized tools, adapted to facilitate the development of the interview in terms of time. Of these tools, 12 items from the Activities of Daily Living Questionnaire (ADLQ) [17] and 7 items from the Dexterity Questionnaire (DextQ-24) [18] were included, making a total of 28 questions (See Supplementary Material S1). The questions were oriented towards 2 points in time, the period prior to the declaration of a global pandemic (11 March 2020) and the current time, 1 year after the pandemic (between 18 January 2021 and 22 March 2021). In this study, and from now on, when we talk about confinement and social restriction, we will refer to the period that lasted the state of alarm decreed by the Government of Spain (between 14 March 2020, and 21 June 2020).

### 2.4. Variables and Data Measurements

Activities of Daily Living Questionnaire (ADLQ) is a tool designed to measure the level of independence in ADLs in clinical outpatient populations, making it a useful measure to quantify the functional capacity of these patients [17]. In this study, we selected 13 items derived from the 6 areas of self-care, home care and management, employment and recreation, shopping and money management, transportation, and communication. The score for each item ranges from 0 to 3, with 0 being no problem and 3 being severe functional impairment [19]. We used ADLQ, which focuses on functional capacity [20] in addition to assessing ADLs in their different modalities and in a more comprehensive manner [20,21].

Dexterity Questionnaire 24 (DextQ-24) is a questionnaire that assesses the degree of difficulty in performing bimanual (12 items) and unimanual (12 items) tasks in functional activities in 5 subgroups: Washing/toileting, dressing, eating and cooking, daily tasks and TV/CD/DVD. The score ranges from 1, no problem, to 4, not being able to perform the activity without external support [18]. In this study, 7 items belonging to the subscales of daily activities and self-care were used.

The selection of ADLQ items was made considering the activities that a patient with PD can usually perform in their daily life since the evaluation allows flexibility in the total score and only considers the activities that are part of the patient’s behavioral repertoire [17]. To this end, in advance of the assessment, an informal interview was held to ask patients what activities they performed regularly. The selection of DextQ-24 items followed the same reasoning. Items that usually form part of the behavioral repertoire of the patient with PD were selected.

### 2.5. Data Analysis

Statistical analysis was performed with SPSS 28.0 for Windows (Copyright© 2013 IBM SPSS Corp., Armonk, NY, USA). For qualitative variables, the number of cases in each category and the corresponding percentage were calculated, and for quantitative variables, the mean and standard deviation were calculated. Normality was tested with the Shapiro–Wilk test. Differences were considered statistically significant at 95% confidence level (*p* < 0.05). To study differences in scores from pre-pandemic to the current time, Wilcoxon tests were performed. Finally, to analyze the correlations between items, Spearman’s correlation coefficient was calculated. The following values were considered regarding the strength of the correlation: rs = [0.00–0.19] (very weak), rs = [0.20–0.39] (weak), rs = [0.40–0.59] (moderate), rs = [0.60–0.79] (strong), rs = [0.80–1.0] (very strong) [22].

Microsoft Excel for Windows, version 2105 (Microsoft^®^ Excel^®^ for Microsoft 365 MSO) was used for the graphs.

## 3. Results

Out of a total of 162 patients with PD interested in participating, the final sample consisted of 126 participants during the interview phase, 36 participants finally declined to participate in the study due to their personal situation (changes in mood due to the confinement situation, low self-esteem and lack of motivation verbally communicated to the interviewers). The sample ranged in age from 36–89 years, 58% were male, and most of them lived with someone else (Table 1).

The change in scores from the pre-pandemic to post-pandemic period was significant for the Basic Activities of Daily Living (BADLs) of bathing, dressing, and eating. Of these, the most affected was dressing, with the mean rising to a score indicating independent but slow or clumsy. The most affected Instrumental Activities of Daily Living (IADLs) were mobility in the community, such as use of public transport and mobility in the neighborhood, as well as cash management and finances, recreational activities and instrumental activities of medication management and home care (See Supplementary Table S1).

**Figure 1:**
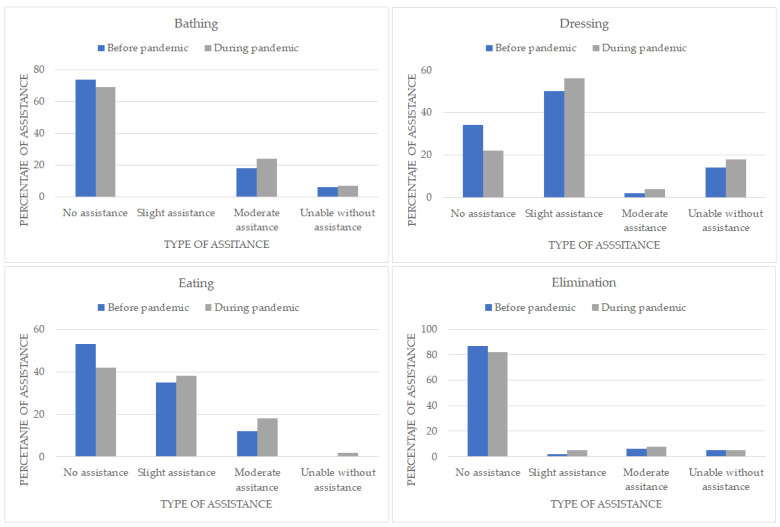
Frequencies of self-care activities.

There were changes in all BADLs compared to before the pandemic: 6% more patients needed moderate assistance with bathing, 1% more patients needed mild assistance with toileting, and 2% more needed moderate assistance. There was a 7% increase in the need for mild assistance with dressing, a 2% increase for moderate assistance, and 4% of patients became incapable without assistance, 4%, 6%, and 1% more patients required mild, moderate, or total assistance with feeding, respectively (Figure 1).

**Figure 2:**
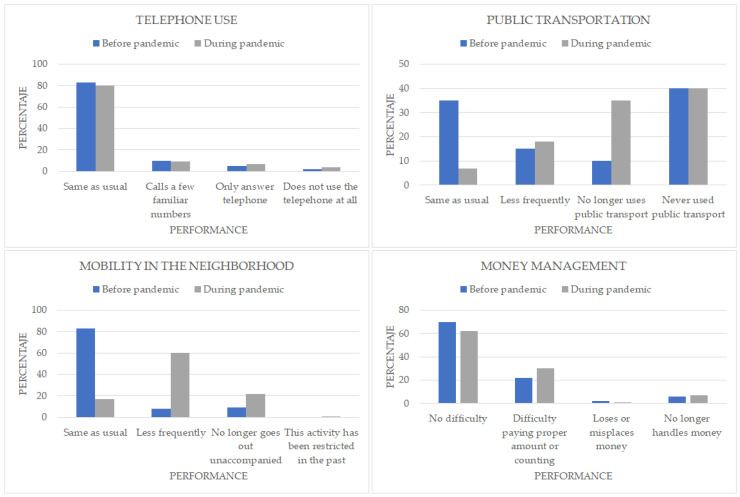
Frequencies of Instrumental Activities of Daily Living (Telephone use, Public Transportation, Mobility in the neighborhood and Money Management).

The proportion of patients requiring assistance with IADLs also increased significantly: 2% switched to not using the telephone and 1% to only answering calls, 27% of patients stopped using public transport regularly, a further 23% stopped using it completely, and 2% used it less frequently. In terms of mobility in the neighborhood, 64% stopped going out as usual, 51% went out less frequently, and a further 13% no longer went out unaccompanied. Regarding cash handling, 10% started to have problems: 8% had difficulties in paying, and 2% stopped handling it, 3% of patients gave up managing their finances. There was a 2% increase in patients needing another person to administer medication and a 3% increase in the need for verbal or written reminders (Figure 2).

**Figure 3:**
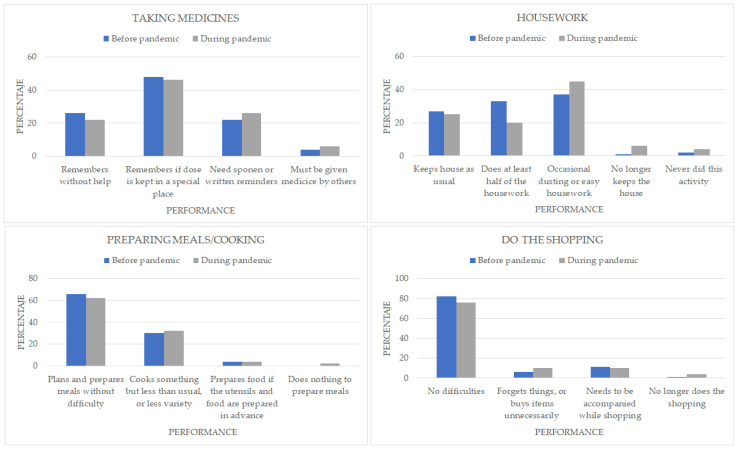
Frequencies of Instrumental Activities of Daily Living (Taking medicines, Housework, Preparing and cooking, Do the Shopping).

Answers to housekeeping questions indicated that 8% more of the participants did small jobs or none at all, and 7% of patients had given up housekeeping compared to before the pandemic situation: 59% had usually maintained their home or done at least half the work, and 45% do now, 3% of participants had started to have difficulties in preparing meals and shopping, 5% more started to forget things, 1% more needed company and 3% stopped going shopping. It should be noted that only the results of those who answered the items referring to home care, food preparation, and shopping have been taken into account: 42% did not answer the item ‘home care’ both PRE and POST, as they did not take care of this task, 55% did not answer the item ‘food preparation’ both PRE and POST, as they did not take care of this task, 46% did not answer the item ‘food shopping PRE’, as they did not deal with this task, 49% did not answer the item ‘shopping for food POST’, as they were not engaged in this task (Figure 3).

Table 2 shows the significant correlations, as well as the level of correlation, between the items of ADLQ and DextQ-24. All the correlations between bathing activity and difficulty in manipulative activities are moderate, especially difficulty in combing the hair, which is almost good (r = 0.579 **), indicating that the greater the assistance required, the greater the difficulty in combing the hair. For elimination, the level of correlation ranges from somewhat related to moderate. For dressing, a large percentage of the correlations are moderate, with a good (r = 0.626 **) association with difficulty in buttoning, i.e., the greater the problem in buttoning, the greater the degree of dependence for dressing. With regard to eating, as for the other activities, most of the correlations are moderate, except for difficulty in eating with a fork, which is good (r = 0.686 **), meaning that the greater the problems in handling a fork, the greater the assistance required with eating.

In terms of IADLs, there is a moderate and positive relationship between telephone use and the ability to dial numbers on the telephone (r = 0.468 **), with associations for the rest of the activities ranging from some association to moderate. It should be noted that mobility in the neighborhood is moderately related to the ability to dial numbers on the phone and meal preparation to the ability to eat with a fork, among others.

All correlations are positive, which is interpreted as the greater the difficulty in manipulative ability in the tasks indicated, the greater the degree of dependence (Table 3).

## 4. Discussion

General population studies, such as Frutos et al. [23], reveal that social isolation resulting from the COVID-19 pandemic has led not only to changes in interpersonal relationships but also in functional capacity and physical activity. This has particularly affected the elderly population, with an increase in functional limitations in their activities of daily living. However, the activities in which the participants report the most limitations vary from those selected in the present study. In the BADLs, they reported going up and down stairs and difficulty getting around, while in our study, they reported bathing, dressing, and eating. In the IADLs, they indicated shopping and preparing meals, while the results of our study show activities such as public transport and mobility in the neighborhood, financial and pharmacological management, and a worsening even in activities that are not carried out outside the home, such as home maintenance, for example, 15% of patients reduced their level of independence in maintaining the home. 

Analyzing the results of our study in detail by percentages and taking into account the categories of assistance, eating was the most impaired activity within the BADLs, with 6% more of the sample requiring moderate assistance, followed by bathing and dressing. Within the IADLs, we found that scores for outdoor mobility showed the greatest variation, with patients basically stating that they did not want to go out unaccompanied, followed by difficulties in handling cash and shopping.

In line with our results, in a study carried out to describe the effects of confinement on the quality of life of patients with PD, Yogev-Seligmann et al. [24] found that a large part of their sample started to experience increased mobility difficulties and required more assistance in general. Our results, in addition to studying the period covered by the confinement and subsequent social isolation measures, are detailed and allow us to observe the causes of the worsening level of independence in all the activities evaluated, as well as the demand for greater assistance and an increase in the human resources required for their performance. The study of Guo et al. [5] also focused on examining the effects of the period of confinement on the quality of life of patients with PD, and their results on the impact of confinement on ADLs were in line with ours. A recently published article on an Italian population [25] also focused only on the period of confinement but found no differences in functionality. These results should be considered in view of the fact that their sample was limited to 12 patients and a 2-month period of confinement. The present study has a larger sample, and our participants suffered from a complete interruption of their rehabilitation services. In contrast to studies published to date focusing only on the period of confinement [8,24,25], our study focuses on a period after 10 to 12 months of social restriction, including confinement and subsequent social restriction measures.

On the other hand, our results show that there is a moderate correlation between the degree of dependence in ADLs and the difficulty in performing activities requiring manipulative dexterity. These findings are interpreted as an increase in dependency due to manipulative difficulty. For example, patients who have greater difficulty in using a spoon, knife, or fork, as determined by the DextQ-24, presented worse functional outcomes not only in feeding but also in bathing or dressing in the ADLQ. In a situation of social isolation such as the one experienced, the use of technology to facilitate communication has increased [26] but, in contrast, 2% of our sample has stopped using the telephone compared to before the pandemic. Moreover, we observed that the greater the difficulty in dialing telephone numbers as measured by the DextQ-24, the lower the use of the telephone as determined by the ADLQ. This suggests that manipulative skills may have hindered the use of this communication resource, further aggravating social isolation.

The pandemic has compromised the usual resources of this population, as almost all patients have had their treatment interrupted and increased inactivity [11] not only during confinement but also during the period after preventive measures of social isolation, as some rehabilitation clinics have remained closed or have assisted telemedically [4,5,11,14,27,28]. Almost all of our sample had no access to rehabilitation services during confinement. Patients in the study by Yogev-Seligmann et al. [24] qualitatively attributed symptom deterioration to the cessation of rehabilitation, as in other studies [4,5,11,14,27,28].

In terms of the methodology used in the present investigation, other studies aimed at finding out how the pandemic had affected PD patients were carried out in a similar way to ours by means of a telephone survey [5,8,29].

Our study is the first to analyze the impact of the pandemic on independence in each of the ADL dimensions of patients with PD, and the results reveal increased functional dependence associated, in turn, with greater difficulty in tasks requiring manipulative dexterity. This provides an overview of the impact caused by social isolation one year after the date on which the COVID-19 pandemic was declared. Our results highlight needs that are likely to be addressed in the daily lives of patients with PD as well as the need for future studies examining the efficacy of intervention programs designed to mitigate these effects. However, further longitudinal studies are needed to investigate the evolution of changes and intensification of the pathological process and worsening of symptoms or ADL performance in patients with PD due to the social isolation caused by the COVID-19 pandemic.

The results of the present study should be interpreted in light of the following limitations. First, this is a cross-sectional design. The results are based solely on information reported by the patients and, therefore, be considered with special caution due to possible biases since this is not an objective measure, nor has the influence that may have been caused by having previously undergone rehabilitative treatment been considered. However, the items are derived from tools that have previously been validated and considered reliable. Moreover, retrospective information is required for pre-confinement questions, which can lead to recall biases [30]. On the other hand, the duration of the disease in the patients included in the study is very heterogeneous (from 1 to 43 years). This is important since the initial stages of PD are characterized by the maintenance of an independent life for most activities, showing only some disturbances in the righting reflexes, quite the opposite in the advanced stages, in which a more significant deterioration occurs. This is a limitation when observing changes in the scores of the evaluation measures, as patients whose disease duration is longer are more likely to have obtained worse results in the evaluations carried out a year later due to the natural progress of the disease and not necessarily because of the isolation produced by the COVID-19.

Despite these limitations, the present study has allowed a first analysis to be made of the possible alterations in the degree of independence in ADLs in PD patients in Spain as a consequence of social isolation due to the global pandemic. This description provides information for designing future interventions at an early stage and, consequently, mitigating the possible adverse effects of the pandemic.

## 5. Conclusions

One year after the start of the social distancing measures imposed due to the COVID-19 pandemic, the participants with Parkinson’s in our sample have worsened their level of dependence in ADLs and IADLs. These results contribute to the fact that the social isolation caused by the pandemic may have been the cause of an increase in the deterioration of manipulative ability, resulting in the loss of ability to perform ADLs. These results show which specific needs will need to be addressed in the rehabilitation of these patients to compensate for the deterioration caused, as well as the design of specific preventive measures to maintain the appropriate level of functionality in the face of possible future situations of social isolation.

## Figures and Tables

**Table 1:** Socio-demographic characteristics of the sample.

	Sample Size N = 126
Age M (SD)	68.03 (10.05)
Range of age (years)	36–89
Sex [N (%)]	
*Male/Female*	73 (58%)/53 (42%)
Disease duration (years) M (SD)	8.89 (7.77)
Place of residence [N (%)]	
*Madrid*	27 (21%)
*Andalucía*	23 (18%)
*Valencia*	23 (18%)
*Extremadura*	20 (16%)
*Galicia*	17 (13%)
*Castilla y León*	9 (7%)
*Canary Islands*	8 (6%)
Caregiver [N (%)]	
*None*	79 (63%)
*Relatives*	28 (23%)
*External caregiver*	15 (12%)
*Other*	4 (3%)
Family living environment [N (%)]	
*Lives with husband or wife*	65 (52%)
*Lives with partner and children / no children*	30 (24%)/7 (6%)
*Alone*	15 (12%)
*Other*	9 (7%)
Rehabilitation treatment prior to pandemic [N (%)]	
*Daycare center*	4 (3.2%)
*Patients’ association*	103 (81.7%)
*Home therapy*	3 (2.4%)
*Others*	7 (5.6%)
*No*	9 (7.1%)
Interruption of rehabilitation treatment during pandemic [N (%)]	
*Yes/No*	125 (99.2%)/1 (0.2%)

**Table 2:** Correlations between ADLQ (BADL’s) and DextQ-24 variables during the alarm state due to the pandemic.

	DextQ-24	Buttoning	Eating	Drinking	Brushing Teeth	Shaving/Making Up	Combing	Dialing a Phone Number
ADLQ (IADL’s)								
**Bathing**		0.532 **	0.454 **	0.541 **	0.332 **	0.393 **	0.579 **	0.460 **
**Elimination**		0.385 **	0.263 **	0.392 **	0.326 **	0.254 **	0.455 **	0.354 **
**Dressing**		0.626 **	0.456 **	0.410 **	0.225 **	0.287 *	0.368 *	0.398 **
**Eating**		0.524 **	0.686 **	0.549 **	0.394 **	0.269 **	0.472 **	0.305 **

Notes: ** denotes significant correlations at the 0.01 level (2-tailed); * denotes significant correlations at the 0.05 level (2-tailed). Bold values are moderate to good correlations.

**Table 3:** Correlations between ADLQ (IADL’s) and DextQ-24 variables during the alarm state due to the pandemic.

	DextQ-24	Buttoning	Eating	Drinking	Brushing Teeth	Shaving/ Making Up	Combing	Dialing a Phone Number
ADLQ (IADL’s)								
**Using telephone**		0.376 **	0.180 *	0.423 **	0.257 **	0.109	0.301 **	0.468 **
**Public transportation**		0.045	0.037	0.021	0.130	0.055	0.088	0.059
**Mobility in neighborhood**		0.443 **	0.290 **	0.394 **	0.225 *	0.312 **	0.341 **	0.408 **
**Handling cash**		0.422 **	0.342 **	0.467 **	0.304 **	0.259 **	0.508 **	0.363 **
**Taking pills**		0.285 **	0.152	0.217 *	0.237 **	0.144	0.171	0.279 **
**Housekeeping**		0.151	0.179	0.196	0.092	0.005	0.007	0.057
**Meal preparation**		0.358 **	0.420 **	0.282 *	0.027	0.093	0.145	0.361 **
**Food shopping**		0.1224	0.372 *	0.258 *	0.201	0.211	0.278 *	0.163

Notes: ** denotes significant correlations at the 0.01 level (2-tailed); * denotes significant correlations at the 0.05 level (2-tailed). Bold values are moderate to good correlations.

## Data Availability

Data sharing is not applicable to this article.

## Supplementary Materials

The following supporting information can be downloaded at: https://www.mdpi.com/article/10.3390/healthcare11121688/s1, Supplementary Table S1: Analysis of differences in ADLQ between the pre-pandemic period and during the pandemic.

## References

[1] Lee S.Y., Kim S.K., Cheon S.M., Seo J.W., Kim M.A., Kim J.W. (2016). Activities of Daily Living Questionnaire from Patients’ Perspectives in Parkinson’s Disease: A Cross-Sectional Study. BMC Neurol..

[2] Sperens M., Georgiev D., Eriksson Domellöf M., Forsgren L., Hamberg K., Hariz G.M. (2020). Activities of Daily Living in Parkinson’s Disease: Time/Gender Perspective. Acta Neurol. Scand..

[3] WHO Director-General’s Opening Remarks at the Media Briefing on COVID-19—11 March 2020. https://www.who.int/director-general/speeches/detail/who-director-general-s-opening-remarks-at-the-media-briefing-on-covid-19---11-march-2020.

[4] Schirinzi T., Di Lazzaro G., Salimei C., Cerroni R., Liguori C., Scalise S., Alwardat M., Mercuri N.B., Pierantozzi M., Stefani A. (2020). Physical Activity Changes and Correlate Effects in Patients with Parkinson’s Disease during COVID-19 Lockdown. Mov. Disord. Clin. Pract..

[5] Santos-García D., Oreiro M., Pérez P., Fanjul G., Paz González J.M., Feal Painceiras M.J., Cores Bartolomé C., Valdés Aymerich L., García Sancho C., Castellanos Rodrigo M. (2020). del M. Impact of Coronavirus Disease 2019 Pandemic on Parkinson’s Disease: A Cross-Sectional Survey of 568 Spanish Patients. Mov. Disord..

[6] Hanff A.M., Pauly C., Pauly L., Schröder V.E., Hansen M., Meyers G.R., Kaysen A., Hansen L., Wauters F., Krüger R. (2021). Unmet Needs of People with Parkinson’s Disease and Their Caregivers During COVID-19-Related Confinement: An Explorative Secondary Data Analysis. Front. Neurol..

[7] Van Der Heide A., Meinders M.J., Bloem B.R., Helmich R.C. (2020). The Impact of the COVID-19 Pandemic on Psychological Distress, Physical Activity, and Symptom Severity in Parkinson’s Disease. J. Park. Dis..

[8] Guo D., Han B., Lu Y., Lv C., Fang X., Zhang Z., Liu Z., Wang X. (2020). Influence of the COVID-19 Pandemic on Quality of Life of Patients with Parkinson’s Disease. Park. Dis..

[9] Brooks S.K., Webster R.K., Smith L.E., Woodland L., Wessely S., Greenberg N., Rubin G.J. (2020). The Psychological Impact of Quarantine and How to Reduce It: Rapid Review of the Evidence. Lancet.

[10] Song J., Ahn J.H., Choi I., Mun J.K., Cho J.W., Youn J. (2020). The Changes of Exercise Pattern and Clinical Symptoms in Patients with Parkinson’s Disease in the Era of COVID-19 Pandemic. Park. Relat. Disord..

[11] Palermo G., Tommasini L., Baldacci F., Del Prete E., Siciliano G., Ceravolo R. (2020). Impact of Coronavirus Disease 2019 Pandemic on Cognition in Parkinson’s Disease. Mov. Disord..

[12] Helmich R.C., Bloem B.R. (2020). The Impact of the COVID-19 Pandemic on Parkinson’s Disease: Hidden Sorrows and Emerging Opportunities. J. Park. Dis..

[13] Subramanian I., Farahnik J., Mischley L.K. (2020). Synergy of Pandemics-Social Isolation Is Associated with Worsened Parkinson Severity and Quality of Life. NPJ Park. Dis..

[14] Brown E.G., Chahine L.M., Goldman S.M., Korell M., Mann E., Kinel D.R., Arnedo V., Marek K.L., Tanner C.M. (2020). The Effect of the COVID-19 Pandemic on People with Parkinson’s Disease. J. Park. Dis..

[15] von Elm E., Altman D.G., Egger M., Pocock S.J., Gøtzsche P.C., Vandenbroucke J.P. (2014). The Strengthening the Reporting of Observational Studies in Epidemiology (STROBE) Statement: Guidelines for Reporting Observational Studies. Int. J. Surg..

[16] Association W.M. (2013). World Medical Association Declaration of Helsinki: Ethical Principles for Medical Research Involving Human Subjects. JAMA.

[17] Johnson N., Barion A., Rademaker A., Rehkemper G., Weintraub S. (2004). The Activities of Daily Living Questionnaire A Validation Study in Patients with Dementia. Alzheimer Dis. Assoc. Disord..

[18] Vanbellingen T., Nyffeler T., Nef T., Kwakkel G., Bohlhalter S., van Wegen E.E.H. (2016). Reliability and Validity of a New Dexterity Questionnaire (DextQ-24) in Parkinson’s Disease. Park. Relat. Disord..

[19] Gleichgerrcht E., Camino J., Roca M., Torralva T., Manes F. (2009). Assessment of Functional Impairment in Dementia with the Spanish Version of the Activities of Daily Living Questionnaire. Dement. Geriatr. Cogn. Disord..

[20] Wicklund A.H., Johnson N., Rademaker A., Weitner B.B., Weintraub S. (2007). Profiles of Decline in Activities of Daily Living in Non-Alzheimer Dementia. Alzheimer Dis. Assoc. Disord..

[21] Durant J., Leger G.C., Banks S.J., Miller J.B. (2016). Relationship between the Activities of Daily Living Questionnaire and the Montreal Cognitive Assessment. Alzheimer’s Dement. Diagn. Assess. Dis. Monit..

[22] Dancey C.P., Reidy J. (2007). Statistics without Maths for Psychology.

[23] Frutos M.L., Cruzado D.P., Lunsford D., Orza S.G., Cantero-Téllez R. (2023). Impact of Social Isolation Due to COVID-19 on Daily Life Activities and Independence of People over 65: A Cross-Sectional Study. Int. J. Environ. Res. Public Health.

[24] Yogev-Seligmann G., Kafri M. (2021). COVID-19 Social Distancing: Negative Effects on People with Parkinson Disease and Their Associations with Confidence for Self-Management. BMC Neurol..

[25] Luis-Martínez R., Di Marco R., Weis L., Cianci V., Pistonesi F., Baba A., Carecchio M., Biundo R., Tedesco C., Masiero S. (2021). Impact of Social and Mobility Restrictions in Parkinson’s Disease during COVID-19 Lockdown. BMC Neurol..

[26] Pišot S., Milovanović I., Šimunič B., Gentile A., Bosnar K., Prot F., Bianco A., Lo Coco G., Bartoluci S., Katović D. (2020). Maintaining Everyday Life Praxis in the Time of COVID-19 Pandemic Measures (ELP-COVID-19 Survey). Eur. J. Public Health.

[27] Feeney M.P., Xu Y., Surface M., Shah H., Vanegas-Arroyave N., Chan A.K., Delaney E., Przedborski S., Beck J.C., Alcalay R.N. (2021). The Impact of COVID-19 and Social Distancing on People with Parkinson’s Disease: A Survey Study. NPJ Park. Dis..

[28] Prasad S., Holla V.V., Neeraja K., Surisetti B.K., Kamble N., Yadav R., Pal P.K. (2020). Parkinson’s Disease and COVID-19: Perceptions and Implications in Patients and Caregivers. Mov. Disord..

[29] Piano C., Bove F., Tufo T., Imbimbo I., Genovese D., Stefani A., Marano M., Peppe A., Brusa L., Cerroni R. (2020). Effects of COVID-19 Lockdown on Movement Disorders Patients With Deep Brain Stimulation: A Multicenter Survey. Front. Neurol..

[30] Zaza S., Wright-De Agüero L.K., Briss P.A., Truman B.I., Hopkins D.P., Hennessy M.H., Sosin D.M., Anderson L., Carande-Kulis V.G., Teutsch S.M. (2000). Data Collection Instrument and Procedure for Systematic Reviews in the Guide to Community Preventive Services. Task Force on Community Preventive Services. Am. J. Prev. Med..

