# Supplementary material for: The Impact of Social Distancing Due to COVID-19 on Activities of Daily Living in Parkinson’s Disease
Source: Healthcare (Basel). 2023 Jun 8;11(12):1688. doi: 10.3390/healthcare11121688 (PMC10298337; doi:10.3390/healthcare11121688)
Supplement: Supplementary file 1 [file healthcare-11-01688-s001.zip › healthcare-2313710-supplementary.pdf]

Supplementary Material S1

INFLUENCIA Y REPERCUSIÓN DEL AISLAMIENTO SOCIAL EN LAS ACTIVIDADES DE LA VIDA DIARIA DE PACIENTES CON ENFERMEDAD DE PARKINSON

Identificación del participante

Número de teléfono:

Entrevistador/a:

En esta entrevista le preguntaremos información necesaria para llevar a cabo este estudio. Si tiene alguna duda o necesita que le aclaremos algo, no dude decirlo. Igualmente, si necesita unos minutos para descansar. Muchas gracias por su participación.

En primer lugar, le pediré información sobre su perfil sociodemográfico, como su edad, personas con las que convive, etc.

1. **Edad:**

2. **Sexo:**
   ☐ Hombre
   ☐ Mujer

3. **Fecha de diagnóstico de la Enfermedad de Parkinson:** __ __ / __ __ / __ __ __ __

4. **Lugar de residencia:**
5. **¿Quiénes son las personas con las que convive?**
   ☐ Solo
   ☐ Sólo con pareja
   ☐ Con pareja más hijos
   ☐ Sólo con hijos
   ☐ Con otros adultos:

6. **¿Quién es su cuidador principal?**
   ☐ No precisa
   ☐ Cónyuge/ pareja
   ☐ Hija
   ☐ Hijo
   ☐ Hermano
   ☐ Hermana
   ☐ Persona externa
   ☐ Otro familiar:

7. **¿Asistía a algún tipo de centro sanitario o recibía algún tipo de terapia (fisioterapia, terapia ocupacional…) antes de la pandemia?**
   ☐ Centro de día
   ☐ Asociación
   ☐ Terapia domicilio
   ☐ Otros:

8. **¿Cesó la actividad en el centro al que acudía o de la terapia que recibía, a causa de la pandemia COVID-19?**
   ☐ No
   ☐ Sí

A. ACTIVIDADES DE LA VIDA DIARIA (AVD):

Instrucciones: ahora, le vamos a preguntar por la capacidad o el grado de dificultad que encuentra al realizar las actividades del día a día.

- **BAÑARSE.**

9. Baño:

Actualmente:

☐ (0) Sin problema
☐ (1) Se baña solo, pero necesita que se lo recuerden.
☐ (2) Se baña solo, con asistencia.
☐ (3) Debe ser bañado por otros.
☐ (9) No lo sé.

Antes de la pandemia (febrero de 2020):

☐ (0) Sin problema
☐ (1) Se baña solo, pero necesita que se lo recuerden.
☐ (2) Se baña solo, con asistencia.
☐ (3) Debe ser bañado por otros.
☐ (9) No lo sé.

- **CUIDADO DEL INTESTINO Y LA VEJIGA.**

10. Evacuación:

Actualmente:

☐ (0) Va al baño independientemente
☐ (1) Va al baño cuando se lo recuerdan; algunos accidentes
☐ (2) Necesita asistencia para la evacuación
☐ (3) No tiene control sobre el intestino o la vejiga
☐ (9) No lo sé

Antes de la pandemia (febrero de 2020):

☐ (0) Va al baño independientemente
☐ (1) Va al baño cuando se lo recuerdan; algunos accidentes
☐ (2) Necesita asistencia para la evacuación
☐ (3) No tiene control sobre el intestino o la vejiga
☐ (9) No lo sé

- **VESTIRSE.**

11. Vestido:

Actualmente:

☐ (0) Sin problema
☐ (1) Independiente, pero lento o torpe
☐ (2) Secuencia incorrecta, olvida pasos
☐ (3) Necesita ayuda para vestirse
☐ (9) No lo sé

Antes de la pandemia (febrero de 2020):

☐ (0) Sin problema
☐ (1) Independiente, pero lento o torpe
☐ (2) Secuencia incorrecta, olvida pasos
☐ (3) Necesita ayuda para vestirse
☐ (9) No lo sé

12. Indique el grado de dificultad para abotonar una camisa, cremalleras o un cinturón:

Actualmente:

☐ (1) Ninguna
☐ (2) Menor
☐ (3) Mayor
☐ (4) Necesita asistencia

Antes de la pandemia (febrero de 2020):

☐ (1) Ninguna
☐ (2) Menor
☐ (3) Mayor
☐ (4) Necesita asistencia

- **ALIMENTACIÓN.**

13. Alimentarse:

Actualmente:

☐ (0) Sin problema
☐ (1) Independiente, pero lento o con algún derrame
☐ (2) Necesita ayuda para cortar o servir; derrama con frecuencia
☐ (3) Debe ser alimentado la mayoría de las comidas
☐ (9) No lo sé

Antes de la pandemia (febrero de 2020):

☐ (0) Sin problema
☐ (1) Independiente, pero lento o con algún derrame
☐ (2) Necesita ayuda para cortar o servir; derrama con frecuencia
☐ (3) Debe ser alimentado la mayoría de las comidas
☐ (9) No lo sé

14. Indique el grado de dificultad para comer con un tenedor, cuchara o cuchillo:

Actualmente:
☐ (1) Ninguna
☐ (2) Menor
☐ (3) Mayor
☐ (4) Necesita asistencia

Antes de la pandemia (febrero de 2020):
☐ (1) Ninguna
☐ (2) Menor
☐ (3) Mayor
☐ (4) Necesita asistencia

15. Indique el grado de dificultad para beber de una taza/vaso:

Actualmente:
☐ (1) Ninguna
☐ (2) Menor
☐ (3) Mayor
☐ (4) Necesita asistencia

Antes de la pandemia (febrero de 2020):
☐ (1) Ninguna
☐ (2) Menor
☐ (3) Mayor
☐ (4) Necesita asistencia

- **HIGIENE Y ARREGLO PERSONAL.**

16. Indique el grado de dificultad para cepillarse los dientes con su cepillo de dientes:

Actualmente:
☐ (1) Ninguna
☐ (2) Menor
☐ (3) Mayor
☐ (4) Necesita asistencia

Antes de la pandemia (febrero de 2020):
☐ (1) Ninguna
☐ (2) Menor
☐ (3) Mayor
☐ (4) Necesita asistencia

17. Indique el grado de dificultad para afeitarse/aplicar maquillaje:

Actualmente:
☐ (1) Ninguna
☐ (2) Menor
☐ (3) Mayor
☐ (4) Necesita asistencia
☐(9) No valorable (no se maquilla/afeita)

Antes de la pandemia (febrero de 2020):
☐ (1) Ninguna
☐ (2) Menor
☐ (3) Mayor
☐ (4) Necesita asistencia
☐(9) No valorable (no se maquilla/afeita)

18. Indique el grado de dificultad para peinar/cepillar el cabello:

Actualmente:
☐ (1) Ninguna
☐ (2) Menor
☐ (3) Mayor
☐ (4) Necesita asistencia

Antes de la pandemia (febrero de 2020):
☐ (1) Ninguna
☐ (2) Menor
☐ (3) Mayor
☐ (4) Necesita asistencia

B. <u>ACTIVIDADES INSTRUMENTALES DE LA VIDA DIARIA:</u>

- **GESTIÓN DE LA COMUNICACIÓN.**

19. Uso del teléfono:

Actualmente:
☐ (0) Igual que siempre
☐ (1) Llama a unos pocos números familiares
☐ (2) Solo atiende el teléfono (no hace llamadas)
☐ (3) No utiliza el teléfono para nada
☐ (9) Nunca tuvo teléfono/No lo sé

Antes de la pandemia (febrero de 2020):
☐ (0) Igual que siempre
☐ (1) Llama a unos pocos números familiares
☐ (2) Solo atiende el teléfono (no hace llamadas)
☐ (3) No utiliza el teléfono para nada
☐ (9) Nunca tuvo teléfono/No lo sé

20. Indique el grado de dificultad marcando los números en el teléfono móvil:

Actualmente:
☐ (1) Ninguna
☐ (2) Menor
☐ (3) Mayor
☐ (4) Necesita asistencia
☐ (9) Nunca tuvo teléfono/No lo sé

Antes de la pandemia (febrero de 2020):

☐ (1) Ninguna
☐ (2) Menor
☐ (3) Mayor

☐ (4) Necesita asistencia
☐ (9) Nunca tuvo teléfono/No lo sé

- **MOVILIDAD EN LA COMUNIDAD.**

21. Transporte público:

Actualmente:

☐ (0) Usa transporte público como habitualmente
☐ (1) Usa transporte público con menos frecuencia
☐ (2) Se ha perdido usando transporte público
☐ (3) Ya no usa transporte público
☐ (9) Nunca usó transporte público regularmente/No lo sé

Antes de la pandemia (febrero de 2020):

☐ (0) Usa transporte público como habitualmente
☐ (1) Usa transporte público con menos frecuencia
☐ (2) Se ha perdido usando transporte público
☐ (3) Ya no usa transporte público
☐ (9) Nunca usó transporte público regularmente/No lo sé

22. Movilidad en el barrio:

Actualmente:

☐ (0) Igual que siempre
☐ (1) Sale con menos frecuencia
☐ (2) Se ha perdido en las inmediaciones del barrio
☐ (3) Ya no sale por el barrio sin compañía
☐ (9) Esta actividad fue restricta en el pasado/No lo sé

Antes de la pandemia (febrero de 2020):

☐ (0) Igual que siempre
☐ (1) Sale con menos frecuencia
☐ (2) Se ha perdido en las inmediaciones del barrio
☐ (3) Ya no sale por el barrio sin compañía
☐ (9) Esta actividad fue restricta en el pasado/No lo sé

- **USO DE LA GESTIÓN FINANCIERA.**

23. Manejo de efectivo:

Actualmente:

☐ (0) Sin problema
☐ (1) Tiene dificultad para pagar montos apropiados, contar
☐ (2) Pierde o traspapela dinero
☐ (3) Ya no maneja dinero
☐ (9) Nunca tuvo responsabilidad en esta actividad/No lo sé

Dificultad para sacar billetes

Antes de la pandemia (febrero de 2020):

☐ (0) Sin problema
☐ (1) Tiene dificultad para pagar montos apropiados, contar
☐ (2) Pierde o traspapela dinero
☐ (3) Ya no maneja dinero
☐ (9) Nunca tuvo responsabilidad en esta actividad/No lo sé

- **GESTIÓN Y MANTENIMIENTO DE LA SALUD.**

24. Tomar la medicación:

Actualmente:

☐ (0) Recuerda sin ayuda
☐ (1) Recuerda tomar la dosis si está en un lugar especial
☐ (2) Necesita recordatorios verbales o escritos
☐ (3) La medicación debe ser administrada por otros
☐ (9) No toma medicación regularmente/No lo sé

Antes de la pandemia (febrero de 2020):

☐ (0) Recuerda sin ayuda
☐ (1) Recuerda tomar la dosis si está en un lugar especial
☐ (2) Necesita recordatorios verbales o escritos
☐ (3) La medicación debe ser administrada por otros
☐ (9) No toma medicación regularmente/No lo sé

- **ESTABLECIMIENTO Y GESTIÓN DEL HOGAR.**

25. Cuidados del hogar:

Actualmente:

☐ (0) Mantiene la casa de manera usual
☐ (1) Realiza al menos la mitad de su trabajo
☐ (2) Limpieza de polvo ocasional o pequeños trabajos
☐ (3) Ya no mantiene la casa
☐ (9) Nunca realizó esta actividad/No lo sé

Antes de la pandemia (febrero de 2020):

☐ (0) Mantiene la casa de manera usual
☐ (1) Realiza al menos la mitad de su trabajo
☐ (2) Limpieza de polvo ocasional o pequeños trabajos
☐ (3) Ya no mantiene la casa
☐ (9) Nunca realizó esta actividad/No lo sé

- **PREPARACIÓN DE LA COMIDA**

26. Preparación de comidas, cocinar:

Actualmente:

☐ (0) Planea y prepara comidas sin dificultad
☐ (1) Algo cocina, pero menos de lo usual, o menos variedad
☐ (2) Prepara la comida si los elementos fueron preparados con anterioridad
☐ (3) No hace nada para preparar comidas
☐ (9) Nunca realizó esta actividad/No lo sé

Antes de la pandemia (febrero de 2020):

☐ (0) Planea y prepara comidas sin dificultad
☐ (1) Algo cocina, pero menos de lo usual, o menos variedad
☐ (2) Prepara la comida si los elementos fueron preparados con anterioridad
☐ (3) No hace nada para preparar comidas
☐ (9) Nunca realizó esta actividad/No lo sé

- **COMPRAS.**

27. Comprar comida:

Actualmente:

☐ (0) Sin problema
☐ (1) Olvida cosas, o compra artículos innecesariamente
☐ (2) Necesita ser acompañado mientras compra
☐ (3) Ya no realiza las compras
☐ (9) Nunca tuvo responsabilidad en esta actividad/No lo sé

Antes de la pandemia (febrero de 2020):

☐ (0) Sin problema
☐ (1) Olvida cosas, o compra artículos innecesariamente
☐ (2) Necesita ser acompañado mientras compra
☐ (3) Ya no realiza las compras
☐ (9) Nunca tuvo responsabilidad en esta actividad/No lo sé

Supplementary Table S1. Analysis of differences in ADLQ between the pre-pandemic period and during the pandemic.

	Before pandemic M(SD)	During pandemic M(SD)	p value
**Bathing**	0.58(1.023)	0.60(1.062)	**0.008**
**Elimination**	0.26(0.761)	0.30(0.793)	0.102
**Dressing**	0.94(0.941)	1.17(0.953)	**0.000**
**Eating**	0.59(0.696)	0.78(0.768)	**0.000**
**Using telephone**	0.25(0.606)	0.30(0.707)	0.109
**Public transportation**	4.15(4.164)	4.85(3.553)	**0.000**
**Mobility in neighbourhood**	0.37(0.901)	1.33(1.213)	**0.000**
**Handling cash**	0.40(0.771)	0.53(0.836)	**0.001**
**Managing finances**	1.94(3.260)	2.02(3.254)	**0.038**
**Taking pills**	1.01(0.764)	1.11(0.792)	**0.008**
**Housekeeping**	1.34(1.510)	1.57(1.552)	**0.002**
**Meal preparation**	2(0.35)	3(0.41)	0.102
**Food shopping**	3(0.32)	3(0.44)	0.140
